# Peritoneal Dissemination and Malignant Ascites in Duodenal Cancer Successfully Treated With Adoptive Cell Therapy Using WT1- and MUC1-Pulsed Dendritic Cells and Activated T Cells With No Adverse Effects: A Case Report

**DOI:** 10.7759/cureus.74834

**Published:** 2024-11-30

**Authors:** Yohsuke Yagawa, Yasunobu Kobayashi, Izumi Fujita, Manabu Watanabe, Shigeo Koido, Haruo Sugiyama, Keishi Tanigawa

**Affiliations:** 1 Department of Immunotherapy, Bio-Thera Clinic, Tokyo, JPN; 2 Department of Surgery, Ebara Hospital, Tokyo, JPN; 3 Department of Surgery, Toho University Ohashi Medical Center, Tokyo, JPN; 4 Internal Medicine, The Jikei University School of Medicine, Tokyo, JPN; 5 Department of Cancer Immunology, Osaka University Graduate School of Medcine, Osaka, JPN

**Keywords:** dendritic cells, duodenal cancer, malignant ascites, muc1, peritoneal dissemination, wt1

## Abstract

A satisfactory treatment for the dissemination of duodenal cancer has not yet been established. We describe a case of peritoneal dissemination and malignant ascites in duodenal cancer that was successfully treated with adoptive cell therapy with no adverse effects. A 72-year-old Japanese male patient with primary duodenal cancer with distal lymph node metastases received chemotherapy with S-1, an oral pyrimidine fluoride*-*derived agent, and oxaliplatin after gastrojejunal bypass, which resulted in tumor shrinkage; however, peritoneal dissemination developed. Despite the administration of a second-line chemotherapy regimen comprising irinotecan, peritoneal dissemination, malignant ascites, and cachexia continued to progress, ultimately resulting in the failure of chemotherapy. He then received adoptive cell therapy with Wilms' tumor 1 (WT1)- and mucin 1 (MUC1) peptide-pulsed dendritic cells (WT1/MUC1-DC) and CD3-activated T lymphocytes (CAT). Following the administration of this treatment eight times per week, the patient’s symptoms and malignant ascites surrounding his cancer disappeared. He developed no adverse effects from this treatment and was able to resume his usual activities without any symptoms. He has continued this treatment every few months as maintenance therapy and has been free of relapse for 54 months. This case suggests a possible beneficial effect of adoptive cell therapy with WT1/MUC1-DC and CAT for peritoneal dissemination and malignant ascites in duodenal cancer.

## Introduction

Duodenal cancer is defined as non-ampullary duodenal malignancy and accounts for only 0.3-0.5% of all gastrointestinal cancers [1,2]. Duodenal cancer is a highly aggressive form of cancer that spreads rapidly within the small intestine. Despite the paucity of data on duodenal cancer, radical resection for resectable duodenal cancer is the only potentially curative option [3]. Chemotherapy is typically used to treat unresectable duodenal cancer. Given the lack of specific chemotherapy regimens for duodenal cancer, those designed for gastric or colorectal cancer are often employed. In the case of malignant ascites, a treatment has not yet been established based on sufficient scientific evidence. Retrospective studies on first-line treatments for unresectable or recurrent duodenal cancer showed that the most commonly used regimen is combination therapy with pyrimidine fluoride and oxaliplatin. Additionally, cisplatin, irinotecan, and gemcitabine have been reported. However, for pyrimidine fluoride plus oxaliplatin combination therapy, median progression-free survival was 6.9-8.2 months and median overall survival was 17.8-22.2 months. In contrast, immune checkpoint inhibitors (ICIs) are strongly recommended for previously treated unresectable or recurrent duodenal cancer with microsatellite instability (MSI) high or mismatch-repair deficient (dMMR) due to their therapeutic effectiveness. Tumors with MSI high or dMMR exhibit increased immunogenicity and have a highly immunostimulated tumor microenvironment (TME) [4]. However, few duodenal cancers have high MSI/dMMR, suggesting that the immune system has not yet fully demonstrated its antitumor potential. Therefore, the development of therapeutic strategies that enhance the antitumor immune system is needed.

Dendritic cells (DCs) are antigen-presenting cells (APCs) that regulate immune responses via interactions between major histocompatibility complex (MHC) molecules on DCs and T cell receptors on T cells, in conjunction with costimulatory molecules. Therefore, DC vaccines may be among the most promising adoptive cell therapy (ACT) options for patients with cancer. However, the low specific antigenicity of cancer cells and the induction of immune tolerance, including immune checkpoints, are considered to reduce this anti-tumor immune response and, consequently, facilitate cancer cell growth [5]. One potential approach to this issue is the loading of synthetic peptides derived from tumor-associated antigens (TAAs) onto DCs for use in therapeutic cancer vaccines. This approach, designated as a DC vaccine, has been utilized in clinical trials involving diverse patient populations afflicted with various types of cancers [5-7]. The National Cancer Institute reported that Wilms' tumor 1 (WT1) and mucin 1 (MUC1) are the most and second most ideal immunogenic TAAs, respectively [8]. Therefore, vaccines using DCs pulsed with WT1 and MUC1 peptides (WT1/MUC1-DC) may represent a viable and minimally invasive treatment option for a range of cancers [9]. In addition, WT1/MUC1-DC are not associated with severe adverse events [10,11].

Another type of ACT is CD3-activated T cell (CAT) therapy. Non-adherent peripheral blood mononuclear cells (PBMCs) may be stimulated by anti-CD3 antibodies, followed by low-dose interleukin-2 (IL-2), which may expand cytotoxic T lymphocytes (CTLs) that are, at least in part, specific for TAAs. CAT returned to a patient’s body to induce antitumor immune responses. Importantly, the combination of CAT with other therapies, such as DC-based vaccines, has the potential to enhance the responsiveness of cancer cells by activating pre-effector CTLs, which are less antigenically expressive and less likely to receive direct activation stimulation without direct antigenic stimulation. A previous study demonstrated that pre-effector CTLs induced by DC vaccines gradually accumulated in peripheral lymphocytes [12]. Therefore, the combination of WT1/MUC1-DC and CAT may enhance the antitumor immune response to duodenal cancer.

We report a case in which the combination of a WT1/MUC1-DC vaccine and CAT was highly efficacious in the treatment of unresectable duodenal cancer with peritoneal dissemination and malignant ascites that had proven refractory to chemotherapy.

## Case presentation

**Figure 1:**
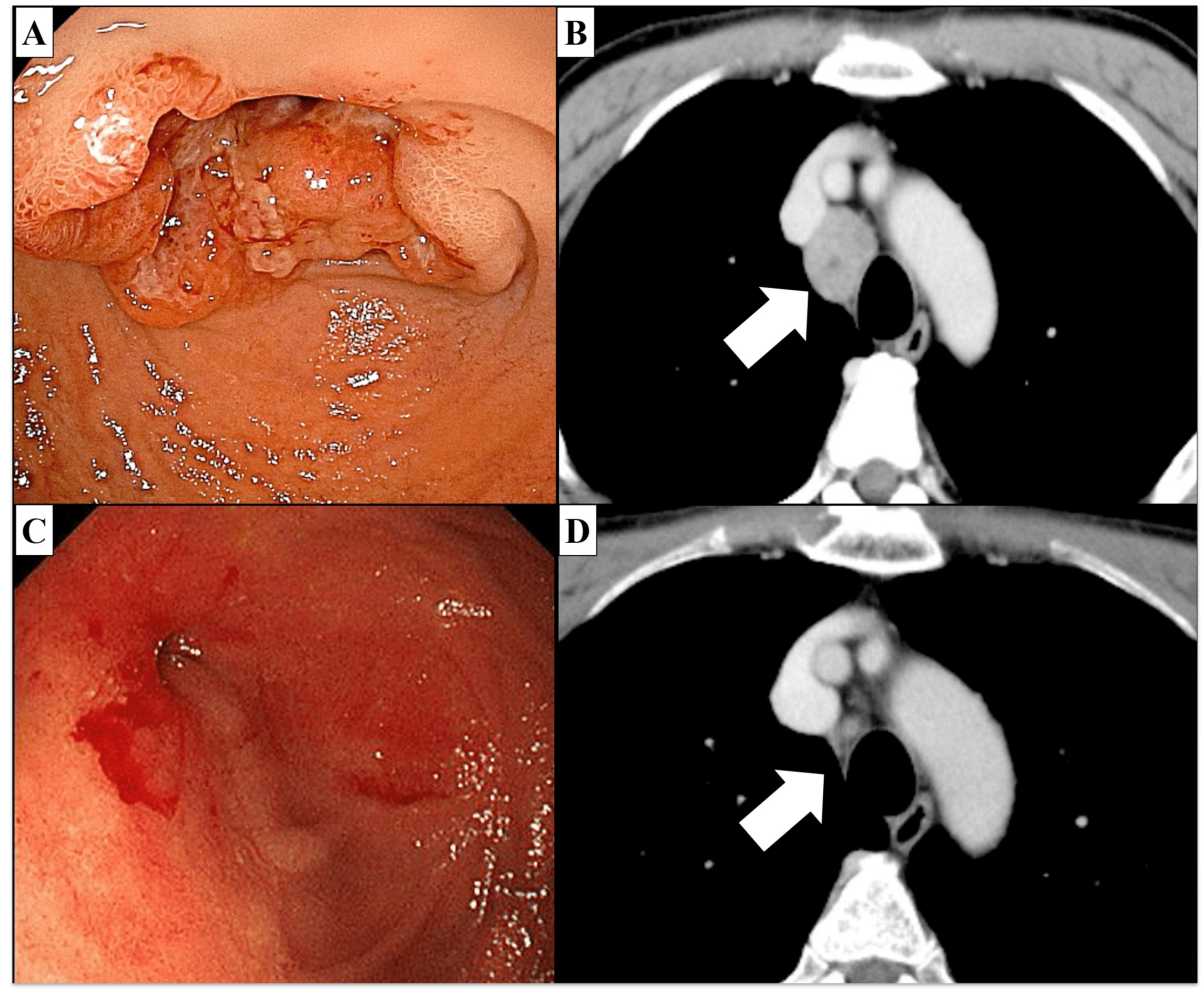
Gastroduodenoscopy and CT taken prior to and following chemotherapy. (A) Gastroduodenoscopy revealed a type 2 circumferential tumor in the descending duodenum. (B) CT imaging showed that the mediastinal lymph node had enlarged to a diameter of 3 cm. (C) Following chemotherapy, gastroduodenoscopy revealed the regression of the duodenal tumor. It was unclear whether the cancer had been entirely eradicated because a cicatricial stricture remained. (D) CT showed a reduction in the size of the mediastinal lymph node.

A 72-year-old Japanese male patient presented to a general hospital with nausea and vomiting. Upon examination, a type 2 tumor extending from the superior duodenal angles to the descending duodenum (Figure 1A) and enlarged lymph nodes in the left supraclavicular and mediastinal region were identified (Figure 1B). A biopsy of the duodenal tumor yielded a diagnosis of adenocarcinoma. A diagnosis of primary duodenal cancer (stage IV: T3N2M1) in accordance with the Union for International Cancer Control, 8th edition was rendered. He underwent gastrojejunal bypass for a duodenal stricture, and chemotherapy with S-1, an oral pyrimidine fluoride-derived agent, and oxaliplatin (SOX) was initiated in June 2019.

**Figure 2:**
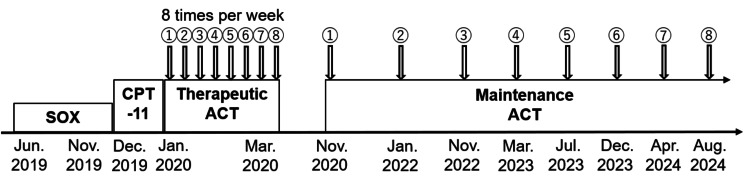
Treatment schedule for chemotherapy and immunotherapy. A patient with primary duodenal cancer with distal lymph node metastases received chemotherapy with SOX between June 2019 and November 2019. A second-line chemotherapy regimen comprising irinotecan (CPT-11) was administered between December 2019 and January 2020. Therapeutic ACT consisted of WT1/MUC1-DC and CAT, which were administered eight times per week between January 2020 and March 2020. The patient was subsequently administered WT1/MUC1-DC and CAT as maintenance therapy from November 2022 onwards, with a frequency of every four to 14 months, resulting in a total of eight treatments. ACT, adoptive cell therapy; WT1, Wilms' tumor 1; MUC1, mucin 1; DC, dendritic cells; CAT, CD3-activated T lymphocytes; SOX, oxaliplatin.

**Figure 3:**
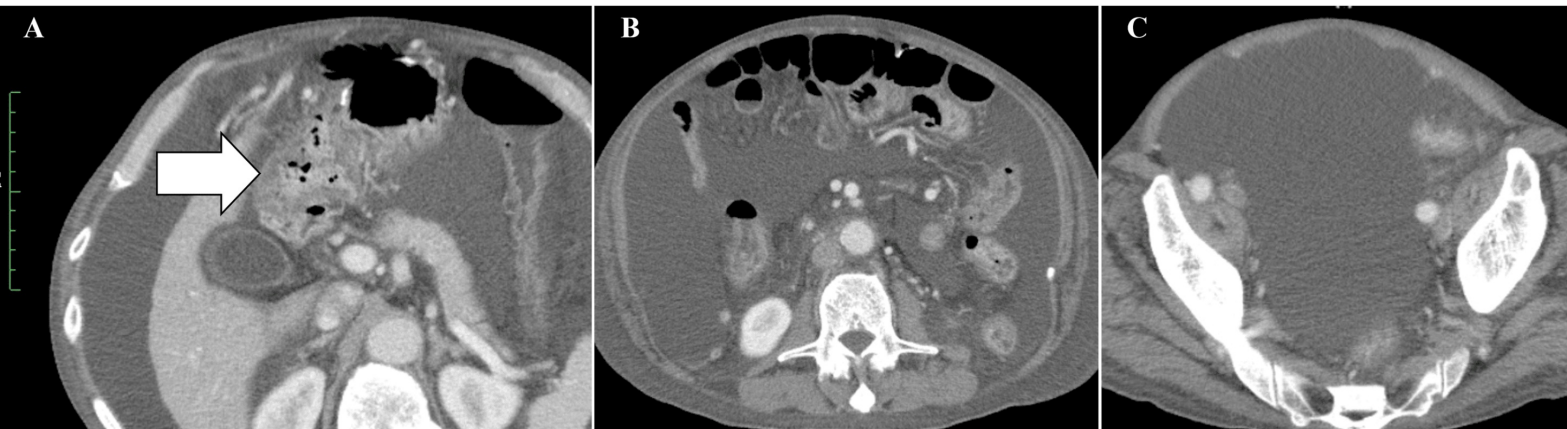
Abdominal CT taken before ACT. (A) A grossly enlarged omentum was observed surrounding the duodenum (arrow). (B, C) A considerable amount of ascites resulting from the dissemination of duodenal cancer was detected. ACT, adoptive cell therapy.

This reduced the size of both the primary lesion and metastatic lymph nodes (Figure 1C, 1D ); however, peritoneal dissemination developed five months after the initiation of SOX chemotherapy. Despite the administration of second-line chemotherapy with irinotecan between December 2019 and January 2020 (Figure 2), peritoneal dissemination, malignant ascites, and cachexia progressed rapidly in the second month of administration (Figure 3). Due to the terminal stage of the disease, a course of palliative care was recommended as the most appropriate treatment option.

The patient subsequently presented at our institution to receive ACT. He had worsening general fatigue, appetite loss (95% less than normal), dysgeusia, diarrhea (7-10 times per day), pathological body weight gain, abdominal bloating, and swelling of both legs. A physical examination yielded the following results: body temperature, 37.3°C; blood pressure, 107/80 mmHg; heart rate, 78 beats/min; and body weight, 67.0 kg. The patient exhibited prominent abdominal distension and edema of the lower extremities despite the administration of diuretic medications. Palpation of the abdominal region revealed the presence of a fluid wave, but neither tenderness nor rebound tenderness. His serum albumin level was 2.3 g/dl (normal range, 3.8-5.2), C-reactive protein was 0.57 mg/dl (normal range, ≤0.30), lactate dehydrogenase was 274 U/l (normal range, 115-245), hemoglobin was 11.9 g/dl (normal range, 13.5-17.6), and his white blood cell count was 2600 /µL (normal range, 3900-9800). Examinations revealed evidence of malnutrition, slight inflammation, and myelosuppression, which were attributed to the progression of cancer and the adverse effects of chemotherapy. Serum levels of CEA and CA19-9 were within the normal ranges. Abdominal computed tomography (CT) revealed a considerable amount of malignant ascites and a dirty fat sign of the omentum around the duodenum (Figure 3).

**Figure 4:**
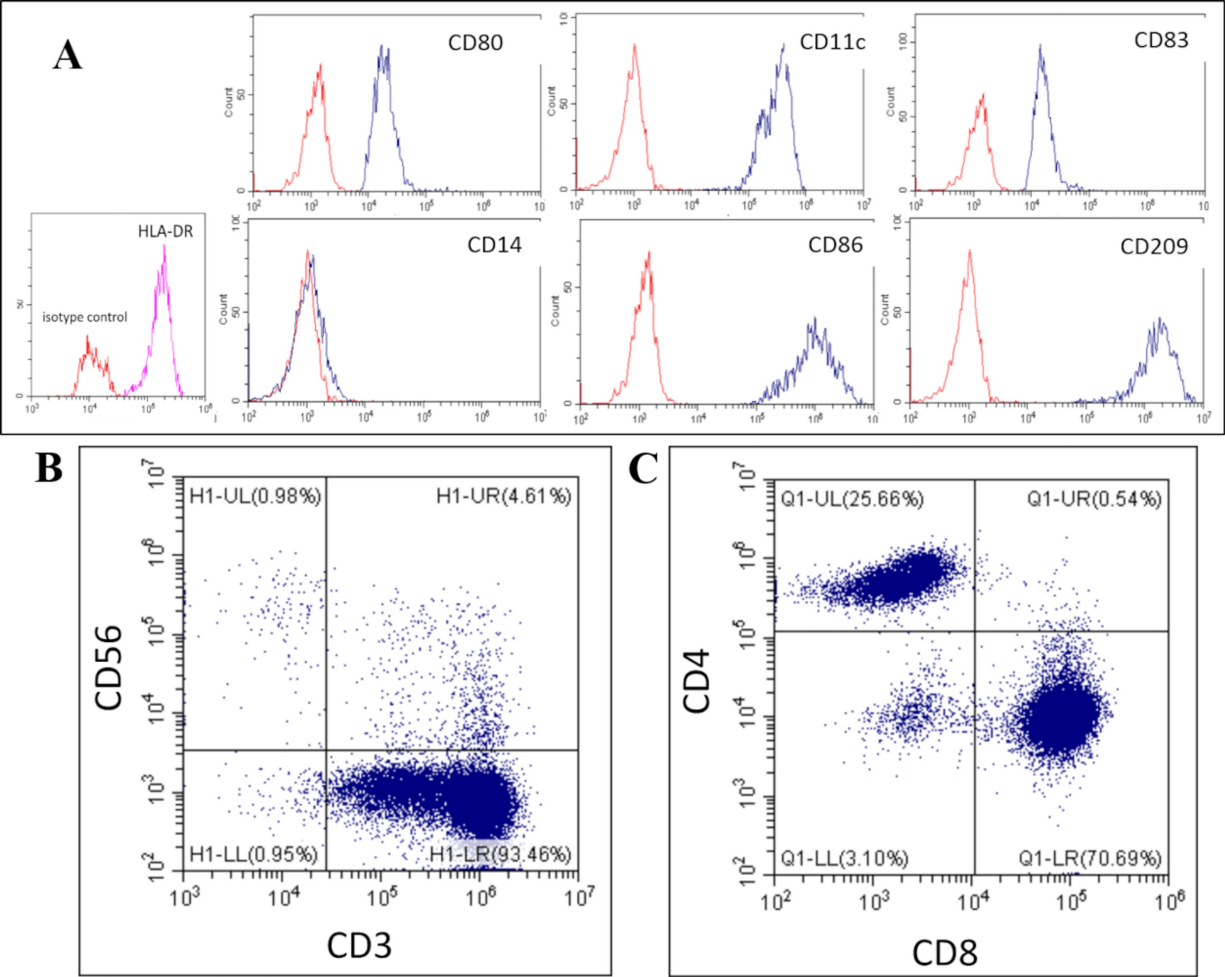
Phenotypic analysis of WT1/MUC1-DC and CAT via a flow cytometric analysis. One representative example of WT1/MUC1-DC is presented. (A) The expressions of HLA-DR, CD11c, CD209, CD80, CD86, CD83, and CD14 on WT1/MUC1-DC is presented. (B) The expression of CD3 and CD56 on CAT is demonstrated. (C) This panel depicts CD4+ and CD8+ cells within the CD3+ cell population in CAT. WT1, Wilms' tumor 1; MUC1, mucin 1; DC, dendritic cells; CAT, CD3-activated T lymphocytes.

In January 2020, ACT was initiated with WT1/MUC1-DC and CAT. In the clinical setting, immunohistochemistry was not employed to select WT1 and MUC1 peptides because previous studies reported the overexpression of WT1 and MUC1 in adenocarcinoma of gastrointestinal cancers [13,14]. The preparation of DCs was conducted in accordance with the previously reported methodology [15]. In brief, PBMCs were collected from the patient using a blood cell separator at each treatment. The PBMC suspension was plated into tissue culture plates and incubated to allow monocytes to adhere to the plastic plate. Immature DCs were harvested from plastic-adherent PBMCs cultured with recombinant human granulocyte-macrophage colony-stimulating factor and recombinant human IL-4. Tumor necrosis factor-alpha was also added to stimulate DCs. The patient’s human leukocyte antigen (HLA)-A types were HLA-A*02:01/02:07. Due to the unavailability of WT1 killer peptides restricted to HLA-A*02:01 or 02:07 for clinical use, only class II binding WT1 helper peptides were employed for the DC vaccination. The WT1 helper peptide (WT134‑51; amino acid sequence, WAPVLDFAPPGASAYGSL) can be used for all MHC class II types. The WT1 helper peptide34‑51 contains a WT1 killer peptide (WT1 killer peptide37‑45; amino acid sequence, VLDFAPPG) that has affinity, at least partially, for HLA‑A*02:01. Moreover, DCs pulsed with the WT134-51 helper peptide elicited not only WT1-specific CD4+ T helper type 1 (Th1) cells but also HLA-A*02:01-restricted CD8+ CTLs in vitro [16]. Therefore, we used the WT1 helper peptide34‑51 to stimulate WT1-specific CD4+ Th1 cells and HLA-A*02:01-restricted CD8+ CTLs simultaneously in this patient. The simultaneous activation of WT1-specific CD4+ Th1 cells and CD8+ CTLs may be essential when treating patients with advanced-stage cancer. On the other hand, a previous study suggested that the MUC1 long peptide (TRPAPGSTAPPAHGVTSAPDTRPAPGSTAP) is applicable to all MHC class I types [14]. The WT134-51 helper peptide and MUC1 long peptide were used to load DCs. To prepare CAT, collected non-adherent PBMCs were stimulated with an immobilized monoclonal antibody to the CD3 antibody and a low dose of recombinant human IL-2 [17,18]. The absence of bacteria, fungi, and endotoxins was subsequently confirmed in both WT1/MUC1-DC and CAT. WT1/MUC1-DC were injected intracutaneously into the patient’s axillary region in an alternating manner, with injections occurring on the right side and then on the left side. CAT was infused intravenously. The patient underwent a course of treatment involving the administration of both cells on a weekly basis for a period of eight consecutive weeks (Figure 2). The mean numbers of WT1/MUC1-DC and CAT for a single administration were 7.5 × 107 and 33.2 × 108, respectively. The mean viability of WT1/MUC1-DC and CAT were 98.1 and 98.8%, respectively. WT1/MUC1-DC were characterized by flow cytometry to ensure that they exhibited the typical phenotype of professional APCs. The phenotype exhibited high levels of HLA-DR, DC markers (CD11c and CD209), costimulatory molecules (CD80 and CD86), a maturation marker (CD83), and low levels of a monocyte and macrophage marker (CD14) (Figure 4A). Furthermore, more than 93% of CAT were CD3+CD56- (Figure 4B), 25.7% were CD4+ CD8-, 70.7% were CD8+CD4-, and 4.6% were CD3+CD56+ (Figure 4C).

**Figure 5:**
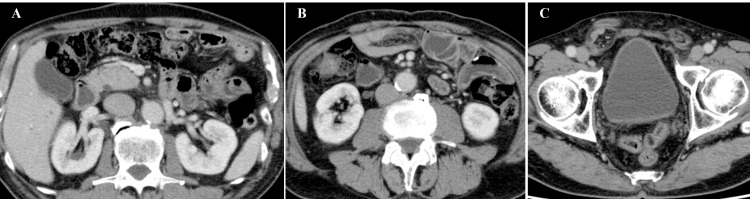
Abdominal CT taken after ACT. (A) The omentum, which is a fatty, viscous tissue located in the abdominal cavity, was no longer visible. (B) Ascites resulting from the dissemination of duodenal cancer had resolved. (C) Right inguinal hernia formed by abdominal pressure caused by massive ascites persisted. ACT, adoptive cell therapy.

It is crucial to assess the efficacy of WT1/MUC1-DC and CAT in inducing WT1- and/or MUC1-specific antitumor immunity. In this case, the induction of antitumor immunity was evaluated in terms of erythema sizes at vaccinated sites. Erythema sizes were quantified 48 hours post-vaccination because they have been shown to correlate with the development of immune responses [15]. The red skin reaction at the injection site of WT1/MUC1-DC appeared after the 4th injection and reached a maximum diameter of 3.5 cm after the eighth injection. Furthermore, although the pretreatment number of peripheral lymphocytes was 500 /µl, it gradually increased and reached 2800 /µl following the administration of CAT eight times. Following the administration of ATC to the patient six times, there was a discernible improvement in his symptoms. It is important to note that one month after the eighth injection, abdominal distention, and leg edema had entirely disappeared and body weight decreased to 57.9 kg, a reduction of 9.1 kg from before therapy. His appetite was fully restored. The patient’s symptoms related to his cancer eventually resolved. Imaging findings of peritoneal dissemination became unclear on CT (Figure 5).

Reductions in the size of the primary lesion and metastatic lymph nodes have been sustained. The patient developed no adverse effects from the treatment and was able to resume his usual activities without any symptoms. To date, after receiving ACT eight times, the patient has continued this therapy, undergoing a total of eight treatments every four to 14 months as maintenance therapy which is conducted to include a restimulation effect for sustaining its efficacy (Figure 2). There has been no evidence of malignant ascites at least 54 months after its administration.

## Discussion

The prognosis in the palliative group of duodenal cancer is poor, with a median survival of five months [19]. It indicates the prognosis of unresectable duodenal cancer with peritoneal dissemination that does not respond to chemotherapy is fatal [20]. A less invasive therapeutic approach with a distinct anticancer mechanism was necessary for this case with cachexia.

DC-loaded antigenic peptides derived from TAAs represent a type of ACT that is designed to facilitate the acquisition of specific immunity against cancer cells that express the corresponding TAAs. Therefore, it is of paramount importance to select the most efficacious TAAs for each case. Clinical studies previously demonstrated the anticancer effects of WT1 peptide [11,21] and MUC1 peptide-loaded DC vaccines [14]. WT1 plays a pivotal role in maintaining the transformation of cancers and tumor escape from immune surveillance. WT1 is expressed not only on the proliferating cells of almost all types of cancers but also on their quiescent stem cells. The eradication of cancer stem cells expressing WT1 that are resistant to chemotherapy and radiotherapy is a critical step in achieving a cure for cancer [13]. MUC1 is a cell membrane glycoprotein that is overexpressed in the majority of tumors and has been implicated in carcinogenesis. It inhibits cell-cell and cell-matrix interactions, preventing epithelial cell aggregation. This is achieved by interfering with integrin-mediated adhesion, which favors detachment from the epithelium, infiltration into the mesenchyme, and the extravasation of cancer cells. Consequently, MUC1 has been also employed as a target for cancer vaccine strategies [22]. It is of significant importance to note that WT1 and MUC1 were identified as the most promising candidates, ranking first and second, respectively [8]. Therefore, the combined use of WT1 and MUC1 as TAAs for DC vaccines is expected to provide coverage for the majority of epithelial cancers. Therefore, the WT1/MUC1-DC vaccine was initially selected for this patient due to the presence of epithelial cancer, which is typically characterized by the overexpression of both WT1 and MUC1.

In the context of DC vaccine settings, WT1 killer peptides that are restricted to HLA-A*24:02 are currently available for clinical use in Japan. However, the patient’s HLA-A types were HLA-A*02:01/02:07, for which WT1 killer peptides were not available. Importantly, the WT134‑51 helper peptide, which can be utilized for all MHC class II types [16], significantly contributes to tumor eradication not only through CD4+ Th1 cells, but also WT1-specific CD8+ CTLs in antitumor immunity [23]. Since the WT1 helper peptide34-51 contains the WT137-45 killer peptide restricted for HLA-A*02:01, DCs loaded with the WT134-51 helper peptide elicit not only WT1-specific CD4+ Th1 cells, but also HLA-A*02:01-restricted CD8+ CTLs. The endogenously processed WT1 helper peptide in DCs has relatively good access to MHC class I and II molecules, which results in the efficient induction of antitumor immunity [16]. The MUC1 long peptide may be utilized for all MHC class I types to induce MUC1-specific immune responses [9,14]. Therefore, the WT134-51 helper peptide and MUC1 long peptide were employed in the present case to induce WT1- and MUC1-specific CTLs. In addition, the WT1/MUC1-DC vaccine was characterized by flow cytometry, and the phenotype exhibited the characteristics of a typical professional APC. Consequently, injected WT1/MUC1-DC may have migrated into the T cell areas of lymph nodes, initiating antigen presentation and inducing WT1/MUC1-T cell responses in this patient.

Furthermore, to achieve an efficacious therapeutic effect, we employed a combination of WT1/MUC1-DC and CAT. The primary objective of ACT is to enhance the number of TAA-specific CTLs. CD3 agonistic antibodies and low-dose IL-2 are frequently employed as tools to expand T cells in a multitude of clinical applications, including adoptive T cell therapy for cancer patients. The utilization of this methodology to expand T cells derived from the patient following vaccination with WT1/MUC1-DC may facilitate the expansion of WT1/MUC1-specific CTLs to some extent. Moreover, the percentage of CD3+CD56+ T cells among all cultured cells for CAT was approximately 4.6% in this patient. CD3+CD56+ T cells are known as killer T cells, which represent a unique population of CTLs with enhanced antitumor activity [24]. The combination of WT1/MUC1-DC and CAT was a potentially efficacious treatment option for this patient. Consequently, ACT may be a viable option for patients with advanced rare cancers who are intolerant of invasive or comprehensive treatments. Moreover, ACT may be employed subsequent to other therapeutic modalities, including chemotherapy and radiation therapy, or as adjuvant therapy following complete remission for the purpose of prophylactic treatment against the development of cancer [15,17]. Some chemotherapeutic agents and radiation therapy induce immunological cell death. There is substantial evidence to show that immunogenic cell death induced by conventional anticancer therapy induces an effective anticancer immune response in the TME as a result of the enhanced therapeutic effects of ACT [13,25]. Consequently, the administration of ACT following chemotherapy proved to be an efficacious intervention in this patient. Another immunological advantage of ACT is the capacity to cultivate immune cells in vitro [26]. However, the anticancer effects of DC vaccines and CAT are contingent upon the patient’s inherent immune status, including their ability to migrate, present antigens, form immunological memory, and attack cancer cells via CTLs, as well as the presence of immunosuppression. Moreover, the heterogeneity of cancer cells results in varying anticancer effects among patients. In the context of current technology, the anticancer effects of ACT are still limited and being developed in terms of knowledge, data, methods, and formulations for treatment. The application of ACT in the earlier stages of cancer is generally preferable due to the numerical advantage that it offers against cancer cells [13,14]. Moreover, the advent of precision medicine, which employs genomic tools, is anticipated to enhance the companion diagnostics and outcomes of ACT [27]. Further studies are needed to elucidate the mechanisms underlying cancer immunology in order to develop ACT.

It was essential to access the induction of antitumor immunity by ACT in this patient. Redness of the skin at the injection site in response to the WT1/MUC1-DC vaccine indicates the presence of a delayed-type hypersensitivity (DTH) immune response, which is caused by the accumulation of memory T cells [9,13,15]. In this case, the diameter reached a maximum of 3.5 cm. A previous study investigating adjuvant therapy in patients with a diameter of 3 cm or more for the DTH reaction demonstrated a more favorable clinical course [15]. Furthermore, the number of peripheral lymphocytes increased to 2800 /µl following the administration of CAT. Flow cytometry with MHC tetramer technology enables the measurement of circulating WT1/MUC1-specific CTLs before and after ACT in some patients [28]. However, MHC tetramers restricted by HLA-A*02:01 were not commercially available.

The combination of ICIs with cancer vaccines may be enticing in light of the necessity for a more detailed understanding of the characteristics of this treatment, and a comprehensive therapeutic strategy for cancer must be devised. In the event that the patient becomes refractory to ATC in the future, it will be necessary to reactivate the anti-tumor effect that had been previously activated by ATC. The combination of ICIs with cancer vaccines may present an attractive avenue for enhancing the immune therapy response, and further clinical studies are warranted.

## Conclusions

The present case demonstrated the anticancer efficacy of ACT in the treatment of peritoneal dissemination and malignant ascites from duodenal cancer, as evidenced by improvements in both survival and quality of life. This treatment was feasible for the patient, even in the terminal phase of cancer, for whom conventional treatment options were exhausted in terms of effectiveness and tolerability. It is important to note that this patient exhibited a positive response to the treatment with no adverse effects. Since awareness of the characteristics of this treatment is required, therapeutic strategies for cancer need to be planned comprehensively.
